# Teaching End-of-Life Decision Making to Undergraduate Nursing Students

**DOI:** 10.1093/geroni/igab046.1266

**Published:** 2021-12-17

**Authors:** Sherry Greenberg, Nancy Innella, Bryan Pilkington

**Affiliations:** 1 Seton Hall University College of Nursing, Seton Hall University College of Nursing, New Jersey, United States; 2 Seton Hall University College of Nursing, Nutley, New Jersey, United States; 3 Seton Hall University, Nutley, New Jersey, United States

## Abstract

This presentation highlights the development, implementation, and results of an educational session with undergraduate nursing students about end-of-life decision making. The purpose of this qualitative thematic analysis study was to explore student perspectives of end-of-life decision making following an education session. The aims were to 1) develop themes from student feedback on end-of-life decision making and 2) refine educational strategies to teach end-of-life decision making to nursing students. The study was conducted with 72 junior level baccalaureate nursing students enrolled in an undergraduate gerontological nursing course. An interactive lecture was developed, following short philosophical ethics readings, which brought the students up to date on the history of end-of-life discussions, key cases, and different frameworks to approach a cluster of ethical issues associated with end-of-life care. A debate pedagogical model was employed as an engaging activity in which students directly applied recently learned concepts. In the debate activity, students were divided into two teams. Each team was assigned a position, which was a specific response to the case question: Should practitioners assist in their patient’s committing suicide? Should practitioners offer medical aid in dying? Each team conferred, presented their position, responded to the arguments or reasons from the opposing position. The session ended with a debrief by the course instructors. In the first semester, 31 nursing students completed four open-ended questions following the class. Results included increased student confidence discussing end-of-life issues and identification of two concepts commonly referred to in end-of-life care discussions and in bioethics, autonomy and dignity.

